# Elevation of Circulating miR-210-3p in High-Altitude Hypoxic Environment

**DOI:** 10.3389/fphys.2016.00084

**Published:** 2016-03-08

**Authors:** Yan Yan, Cheng Wang, Wanqing Zhou, Yonghui Shi, Pengtao Guo, Yuxiu Liu, Junjun Wang, Chen-Yu Zhang, Chunni Zhang

**Affiliations:** ^1^Department of Clinical Laboratory, Jinling Hospital, State Key Laboratory of Analytical Chemistry for Life Science, Jiangsu Engineering Research Center for MicroRNA Biology and Biotechnology, Nanjing University School of Medicine, Nanjing UniversityNanjing, China; ^2^State Key Laboratory of Pharmaceutical Biotechnology, Collaborative Innovation Center of Chemistry for Life Sciences, Jiangsu Engineering Research Center for MicroRNA Biology and Biotechnology, NJU Advanced Institute for Life Sciences, School of Life Sciences, Nanjing UniversityNanjing, China; ^3^Department of Clinical Laboratory, The Affiliated Hospital of Nanjing University Medical School, Nanjing Drum Tower Hospital, Nanjing UniversityNanjing, China; ^4^Department of Clinical Laboratory, The Forty-First Hospital of PLANêdong, China; ^5^Department of Medical Statistics, Nanjing University School of Medicine, Jinling Hospital, Nanjing UniversityNanjing, China

**Keywords:** high-altitude-hypoxia, Tibet, Tibetan, Han Chinese, miR-210-3p

## Abstract

**Background:** The induction of miR-210-3p, a master hypoxamir, is a consistent feature of the hypoxic response in both normal and malignant cells. However, whether miR-210-3p acts as a circulating factor in response to a hypoxic environment remains unknown. The current study aimed to examine the effect of a high-altitude hypoxic environment on circulating miR-210-3p.

**Methods:** We examined and compared the levels of miR-210-3p using TaqMan-based qRT-PCR in both peripheral blood cells and plasma from 84 ethnic Chinese Tibetans residing at 3560 m, 46 newly arrived migrant Han Chinese (Tibet Han) and 82 Han Chinese residing at 8.9 m (Nanjing Han). Furthermore, we analyzed the correlations of miR-210-3p with hematological indices.

**Results:** The relative concentrations of miR-210-3p to internal reference U6 in blood cells were significantly higher in the Tibet Han group (1.01 ± 0.11, *P* < 0.001) and in the Tibetan group (1.17 ± 0.09, *P* < 0.001) than in the Nanjing Han group (0.51 ± 0.04). The absolute concentrations of plasma miR-210-3p were also markedly elevated in the Tibet Han group (503.54 ± 42.95 fmol/L, *P* = 0.004) and in the Tibetan group (557.78 ± 39.84 fmol/L, *P* < 0.001) compared to the Nanjing Han group (358.39 ± 16.16 fmol/L). However, in both blood cells and plasma, miR-210-3p levels were not significantly different between the Tibet Han group and the Tibetan group (*P* = 0.280, *P* = 0.620, respectively). Plasma miR-210-3p concentrations were positively correlated with miR-210-3p levels in blood cells (*r* = 0.192, *P* = 0.005). Furthermore, miR-210-3p levels in both blood cells and plasma showed strong positive correlations with red blood cell counts and hemoglobin and hematocrit values.

**Conclusion:** These data demonstrated, for the first time, that miR-210-3p might act as a circulating factor in response to hypoxic environments and could be associated with human adaptation to life at high altitudes.

## Introduction

MicroRNAs (miRNAs), which are endogenously expressed small noncoding RNAs, regulate gene expression at the post-transcriptional level by either degrading or blocking the translation of messenger RNA targets. Recent studies have strongly suggested that miRNAs act as critical mediators of the hypoxic response (Yan et al., 2009; Nallamshetty et al., 2013; Rupaimoole et al., 2014). miR -210 represents a major hypoxia-inducible miRNA also known as a hypoxamir, which is ubiquitously expressed in a broad range of cells (Huang et al., 2010; Chan et al., 2012; Huang and Zuo, 2014; Qu et al., 2014). Emerging evidence has demonstrated that the induction of miR-210-3p, as a robust target of hypoxia-inducible factors (HIFs), is a consistent feature of the hypoxic response in both normal and malignant cells, and its overexpression has been detected in a variety of diseases with hypoxic components (Devlin et al., 2011; Kelly et al., 2011; Cicchillitti et al., 2012; Hale et al., 2014; Qu et al., 2014; Wang et al., 2014). miR-210-3p has been reported as the master regulator of hypoxic tumor response in various cancers (Huang et al., 2009; Greither et al., 2010) by disturbing mitosis through targeting multigenes involved in mitotic progression (He et al., 2013). More importantly, miR-210-3p was identified as deeply involved in the erythroid phenotype. Several functions related with erythroid cells were demonstrated to be regulated by miR-210-3p, including maturation and proliferation of early erythroid cells, expression of fetal g-globin genes and enucleation (Kosaka et al., 2008; Bianchi et al., 2009). Recently, our group and others have discovered that miRNAs are stably secreted into the bloodstream, as well as in response to tissue injury and other pathological conditions (Chen et al., 2008; Lawrie et al., 2008; Mitchell et al., 2008; Zhang et al., 2010; Wang et al., 2015; Wronska et al., 2015). Increases in the plasma-based expression of miR-210-3p have been reported in the context of various cancers and other hypoxia-induced pathological conditions (Raitoharju et al., 2011; Greco et al., 2012; Whitehead et al., 2013; Huang and Zuo, 2014; Ono et al., 2015). However, whether miR-210-3p might act as a circulating factor in response to hypoxic environments has barely been explored.

High-altitude hypoxia is caused by decreased barometric pressure, which results in reduced arterial oxygen content that, therefore, exerts formidable physiological stress on the human body. The extreme environmental conditions experienced at high altitudes challenge the ability of humans to adapt and/or acclimatize. Elevated erythropoietin (EPO), erythrocytes, and hemoglobin (HGB) and increased angiogenesis are common responses to hypoxic stress, which allow for more efficient oxygen utilization. Individuals from low-altitude populations (such as the Han Chinese) who move to live at higher altitudes suffer from a number of potentially lethal diseases specifically related to the low levels of oxygen (Wu and Miao, 2001; Wu, 2004; León-Velarde et al., 2005), and they often struggle to reproduce at these altitudes Moore et al., 2001a,b; Julian et al., 2009, whereas populations living in high-altitude regions, such as the Tibetan people of Chinese Tibet, exhibit a unique suite of physiological changes, including circulatory, respiratory, and hematological adaptations to life with decreased available oxygen at high altitudes (Ge et al., 1994; Chen et al., 1997; Beall, 2007). Genetic selection for high altitude adaptation and variations in single nucleotide polymorphisms between Tibetans and lowlander populations have been extensively documented (Beall, 2007; Simonson et al., 2010; Yi et al., 2010). An attenuated response to low oxygen, lower levels of erythrocyte, increased blood flow due to reduced viscosity of the blood and changes in respiratory physiology correlate well with genetic selection. However, very little research has been devoted to the identification of the miRNAs responsible for the adaptation/acclimatization to high altitude hypoxia.

Therefore, in the present study, we examined and compared the expression levels of miR-210-3p in both plasma and peripheral blood cells samples from Han Chinese subjects residing in Nanjing, which lies at sea level (Nanjing Han), Han Chinese who have recently immigrated from the plains to placeTibet (Tibet Han) and ethnic Chinese Tibetans. We also analyzed the correlations between miR-210-3p and hematological indices.

## Methods

### Study design and subjects

The present study enrolled 84 healthy, native Tibetans volunteers who resided in Nêdong, a county of Lhoka prefecture in the Tibet Autonomous Region, at an altitude of 3560 m (Tibetan), 46 ethnic Han Chinese who immigrated 1–2 years ago (mean of 17 months) from the plains of East China to Nêdong (Tibet Han) and 82 Han Chinese residing in Nanjing (Nanjing Han), a city located in East China, at an altitude of 8.9 m. The Tibet Han and Tibetan participants were recruited from among individuals reporting for routine health checkups at the Forty-First Hospital of the PLA in Nêdong, China, and the Nanjing Han participants were recruited from among individuals who had visited the Jinling Hospital in Nanjing, China, for routine checkups between 2013 and 2014. All of the participants were healthy.

All of the blood samples were collected in EDTA tubes using a standard operating procedure. Written informed consent was obtained from all of the participants prior to the study. The study protocol was approved by the ethics committees of Jinling Hospital and the Forty-First Hospital of PLA. The study was performed in accordance with the Declaration of Helsinki.

### Hematology and plasma EPO analysis

Hematological indices, including red blood cell count (RBC), HGB and hematocrit (HCT), were measured with XE-2100 analyzers (Sysmex, Kobe, Japan) with commercial reagents, immediately after drawing a venous blood sample. Plasma EPO concentration was measured with commercial enzyme-linked immunosorbent assay (ELISA) kits (Cusabio, Wuhan, China).

### RNA isolation and qRT-PCR assay of miR-210-3p in plasma and peripheral blood cells

RNA from the plasma and peripheral blood cell samples was prepared for qRT-PCR assay at roughly the same time. Total RNA was extracted from 100 μL of plasma with a 1-step phenol/chloroform purification protocol, as previously described (Wang et al., 2015). A TaqMan probe-based qRT-PCR assay of plasma miR-210-3p was conducted on a 7300 Sequence Detection System (Applied Biosystems, Foster City, CA, USA) according to the manufacturer's instructions, with a minor modification as described previously (Zhang et al., 2010). All of the reactions were run in triplicate, and the average Cq values were calculated. To control for variability in the RNA extraction and purification procedures, an exogenous reference gene, plant miRNA MIR2911 (5′-GGCCGGGGGACGGGCUGGGA), was spiked into each plasma sample at a final concentration of 10^6^ fmol/L during RNA isolation. The Cq values of MIR2911 remained constant among the three studied groups (Figure S1). Relative concentrations of miR-210-3p were normalized to MIR2911 and were calculated using the comparative Cq method (2^−ΔCq^). ▵ Cq was calculated by subtracting the Cq values of MIR2911 from the Cq values of the miR-210-3p. The relative concentration of miR-210-3p to MIR2911 was calculated using the equation 2^−ΔCq^. Furthermore, we calculated the absolute concentration of miR-210-3p from calibration curve developed with corresponding synthetic single-strand miR-210-3p oligonucleotide as previously described (Liu et al., 2011, 2012; Chen et al., 2012; Luo et al., 2013). The standard curve was prepared by ten-fold serial dilution of synthetic miR-210-3p oligonucleotide (TaKaRa, Dalian, China) from 100 fmol/L to 10^3^ pmol/L, and the level of this miR-210-3p oligonucleotide was assessed by qRT-PCR assay. The resulting Cq values were plotted vs. the log_10_ of the amount of the synthetic miR-210-3p (Figure S2). Each sample and each dilution of the calibrator were run in triplicate for analysis.

The total RNA of peripheral blood cells was extracted with TRIzol Reagent (Invitrogen, Carlsbad, CA, USA), according to the manufacturer's instructions. The concentration and quality of the extracted RNA were determined with a spectrophotometer (Eppendorf, Hamburg, Germany) at 260 nm and 280 nm. U6 was used as an internal reference for the qRT-PCR analysis of miR-210-3p in peripheral blood cells because its Cq values did not show differences among the three studied groups (Figure S3). The relative content of miR-210-3p was normalized to U6 and was calculated using the comparative Cq method (2^−ΔCq^) with the same method described above.

### Data analysis

All of the statistical analyses were performed using the SPSS software, version 17.0. The concentrations of miR-210-3p and EPO are presented as the mean ± SEM, and other variables are expressed as the mean ± SD. The differences in variants among groups were analyzed by one-way ANOVA, and the differences between groups were subsequently determined by the nonparametric Mann-Whitney U-test. The two-sided x^2^ test was used to compare sex distributions between two groups. A *P* < 0.05 was considered statistically significant. Spearman's rank correlation analysis was conducted to analyze correlations between variables.

## Results

### Demographic and hematological features of all participants

The demographic and hematological features of the three studied groups are summarized in Table 1. There were no significant differences in age or sex distributions among the Nanjing Han, Tibet Han and Tibetan groups. The RBC, HGB, and HCT values of both men and women, however, were significantly increased (*P*-values ranging from < 0.05 to < 0.001) in the Tibet Han group compared with the Nanjing Han group. The HGB and HCT values of both men and women (*P* = 0.001), along with the women's RBC values (*P* = 0.02), were also significantly higher in the Tibetan group than in the Nanjing Han group. Furthermore, we compared these parameters between the Tibet Han group and the Tibetan group, and observed that the RBC, HGB, and HCT values were significantly increased in the Tibet Han men compared to the Tibetan men (*P* = 0.005, *P* = 0.014, and *P* = 0.024, respectively). Similarly, the levels of RBC, HGB, and HCT were also higher in the Tibet Han women than in the Tibetan women; however, these differences did not achieve statistical significance. In addition, we evaluated the concentrations of plasma EPO in some of the studied individuals. As a result, the plasma concentrations of EPO in both men and women were also significantly increased in the Tibet Han group and the Tibetan group compared with the Nanjing Han group (*P* < 0.05); however, the EPO concentration was not different between the Tibet Han group and the Tibetan group (Table 1).

**Table 1:** **Demographic and hematological features of the Nanjing Han, Tibet Han, and Tibetan groups^a^**.

**Variable**	**Nanjing Han**	**Tibet Han**	**Tibetan**	***P*^b^**	***P*^c^**	***P*^d^**
n	82	46	84			
Sex				0.142^e^	0.163^e^	0.763^e^
Male	37	27	47			
Female	45	19	37			
Age, years	33.25±9.06	33.00±10.57	35.47±10.07	0.892	0.170	0.146
Male	34.59±7.64	32.11±10.15	36.42±10.58	0.309	0.387	0.065
Female	31.98±9.97	34.26±11.29	34.30±9.27	0.405	0.298	0.991
**HEMATOLOGICAL INDICES**						
RBC (10^12^/L)	4.73±0.45	5.31±0.70	4.86±0.71	< 0.001	0.404	0.002
Male	5.08±0.34	5.59±0.67	5.01±0.85	0.003	0.931	0.005
Female	4.44±0.28	4.90±0.53	4.67±0.43	0.007	0.02	0.254
HGB (g/L)	138.73±13.82	170.33±21.85	158.63±21.85	< 0.001	< 0.001	0.032
Male	150.00±10.75	179.15±19.73	164.38±21.65	< 0.001	0.001	0.014
Female	129.47±7.90	157.79±18.68	145.92±15.51	< 0.001	< 0.001	0.077
HCT (%)	38.28±6.57	50.31±6.60	46.58±6.59	< 0.001	< 0.001	0.002
Male	39.46±7.88	52.92±6.31	48.49±7.31	< 0.001	< 0.001	0.024
Female	37.31±5.15	46.61±5.20	43.60±34.26	< 0.001	< 0.001	0.128
EPO (mIU/ml)	95.52±5.97	159.11±13.87	184.07±14.74	< 0.001	< 0.001	0.350
n (male/female)	60(24∕26)	40(21∕19)	50(28∕22)	0.461^e^	0.548^e^	0.832^e^
Male	123.66±10.78	191.12±18.51	178.00±18.75	0.010	0.047	0.946
Female	60.32±6.08	130.60±19.42	191.78±23.92	0.001	< 0.001	0.063

*RBC, red blood cell counts; HGB, hemoglobin; HCT, hematocrit; EPO, erythropoietin*.

a *Data are presented as mean ± standard deviation, EPO are presented as mean ± standard error*.

b *Tibet Han vs. Nanjing Han*;

c *Tibetan vs. Nanjing Han*;

d *Tibetan vs. Tibet Han*.

e *Two-sided χ^2^ test*.

### Plasma miR-210-3p concentrations

We measured the concentrations of miR-210-3p in plasma samples from all of the participants by qRT-PCR assay. The relative concentrations of miR-210-3p to MIR2911 were markedly elevated in the Tibet Han and Tibetan groups as compared with the Nanjing Han group (*P* < 0.001) (Figure S4). The plasma absolute concentrations of miR-210-3p were also significantly higher in the Tibet Han and Tibetan groups than in the Nanjing Han group (*P* = 0.004, *P* < 0.001, respectively) (Table 2). However, no marked difference in plasma miR-210-3p concentration was observed between the Tibet Han group and the Tibetan group (*P* = 0.620).

**Table 2:** **The concentrations of miR-210-3p in plasma and peripheral blood cells from Nanjing Han, Tibet Han, and Tibetan determined by qRT-PCR assay^a^**.

**miR-210-3p**	**Nanjing Han**	**Tibet Han**	**Fold change^b^**	***P*^b^**	**Tibetan**	**Fold change^c^**	***P*^c^**	***P*^d^**
Plasma	358.39 ± 16.16	503.54 ± 42.95	1.41	0.004	557.78 ± 39.84	1.56	< 0.001	0.620
Blood cells	0.51 ± 0.04	1.01 ± 0.11	1.98	< 0.001	1.17 ± 0.09	2.29	< 0.001	0.280

a *The absolute concentration of miR-210-3p in plasma is presented as the mean ± SEM (fmol/L), and the relative concentration of miR-210-3p to U6 in peripheral blood cells is presented as the mean ± SEM*.

b *Tibet Han/Nanjing Han*.

c *Tibetan/Nanjing Han*.

d *Tibetan/Tibet Han. FC, fold change*.

### Expression level of miR-210-3p in peripheral blood cells

Furthermore, we determined miR-210-3p expression levels in peripheral blood cells using qRT-PCR assay. The results of miR-210-3p in blood cells were in agreement with those of plasma samples, showing that the expression level of miR-210-3p was markedly increased in the Tibet Han and Tibetan groups compared to the Nanjing Han group (1.98-fold increase, *P* < 0.001; 2.29-fold increase, *P* < 0.001, respectively) (Table 2). Similarly, the miR-210-3p level was also higher in the Tibetan group than in the Tibet Han group; however, the difference did not achieve statistical significance (*P* = 0.280).

### Correlation between plasma miR-210-3p and miR-210-3p in peripheral blood cells

**Figure 1:**
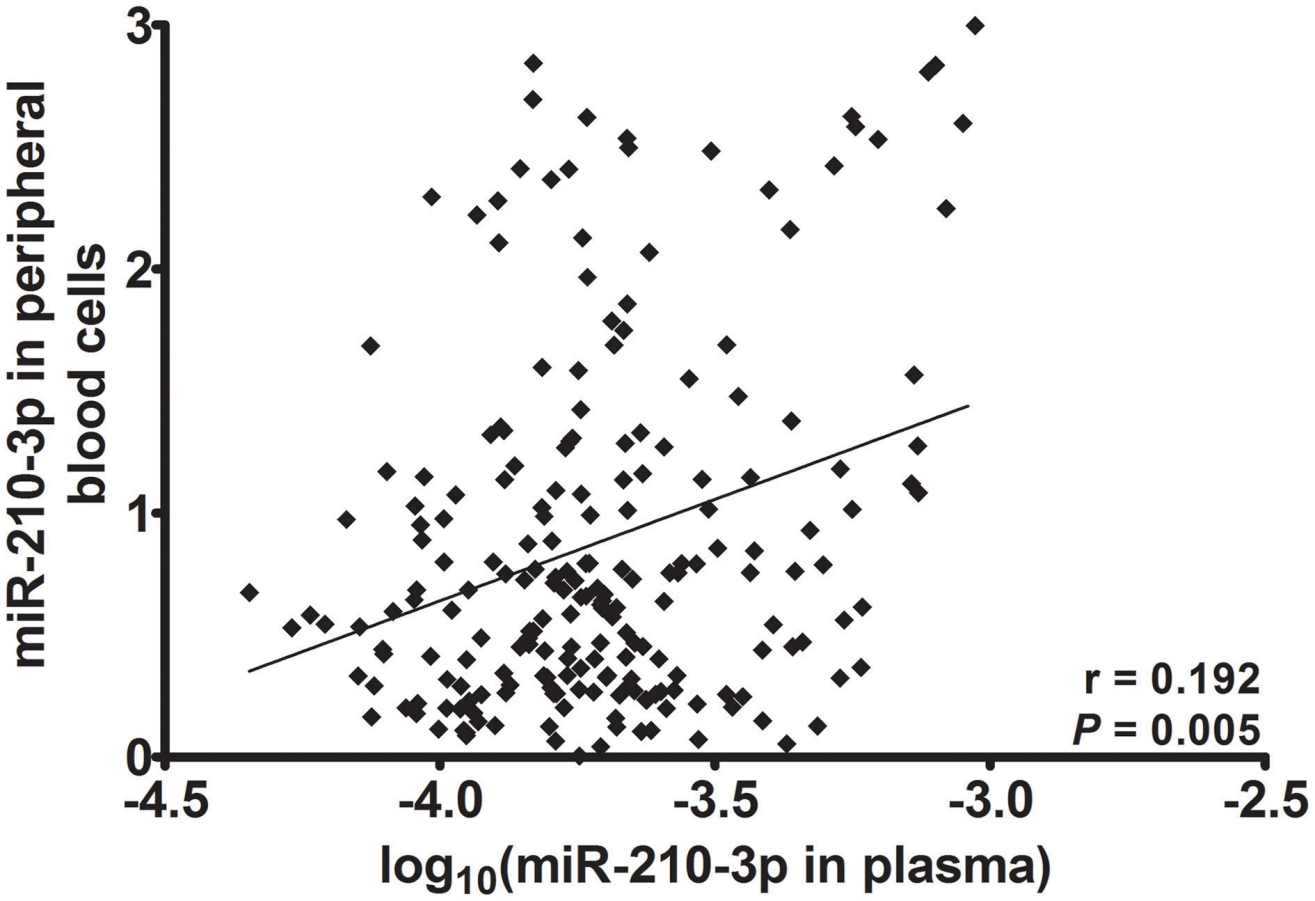
**Spearman's rank correlation between miR-210-3p in plasma and in peripheral blood cells**.

Subsequently, we used Spearman's rank correlation analysis to test for the correlations between the relative concentrations of miR-210-3p to MIR2911 in plasma and relative concentrations of miR-210-3p to U6 in blood cell in all of the studied individuals. As shown in Figure 1, a positive correlation was observed between the two (r = 0.192, *P* = 0.005). This result indicated that plasma miR-210-3p might partly originate from peripheral blood cells.

### Relationships of miR-210-3p with hematological indices and EPO

**Figure 2:**
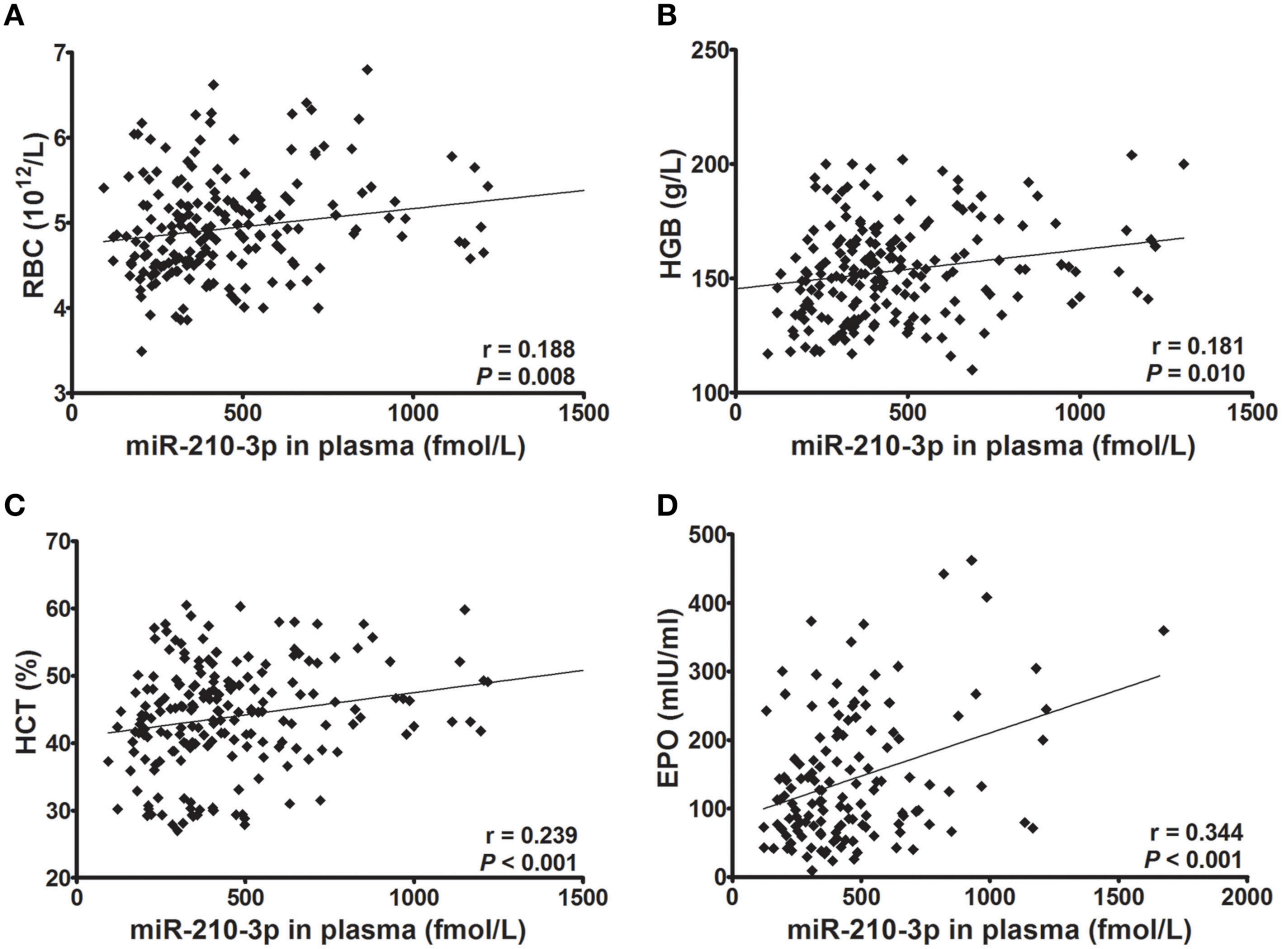
**The correlations of absolute concentrations of plasma miR-210-3p with hematological indices and plasma EPO concentrations (***n*** = 212). (A–D)** Correlations of plasma miR-210-3p concentrations with red blood cell count (RBC) and with hemoglobin (HGB), hematocrit (HCT), and plasma erythropoietin (EPO) values, calculated by Spearman's rank correlation analysis.

**Figure 3:**
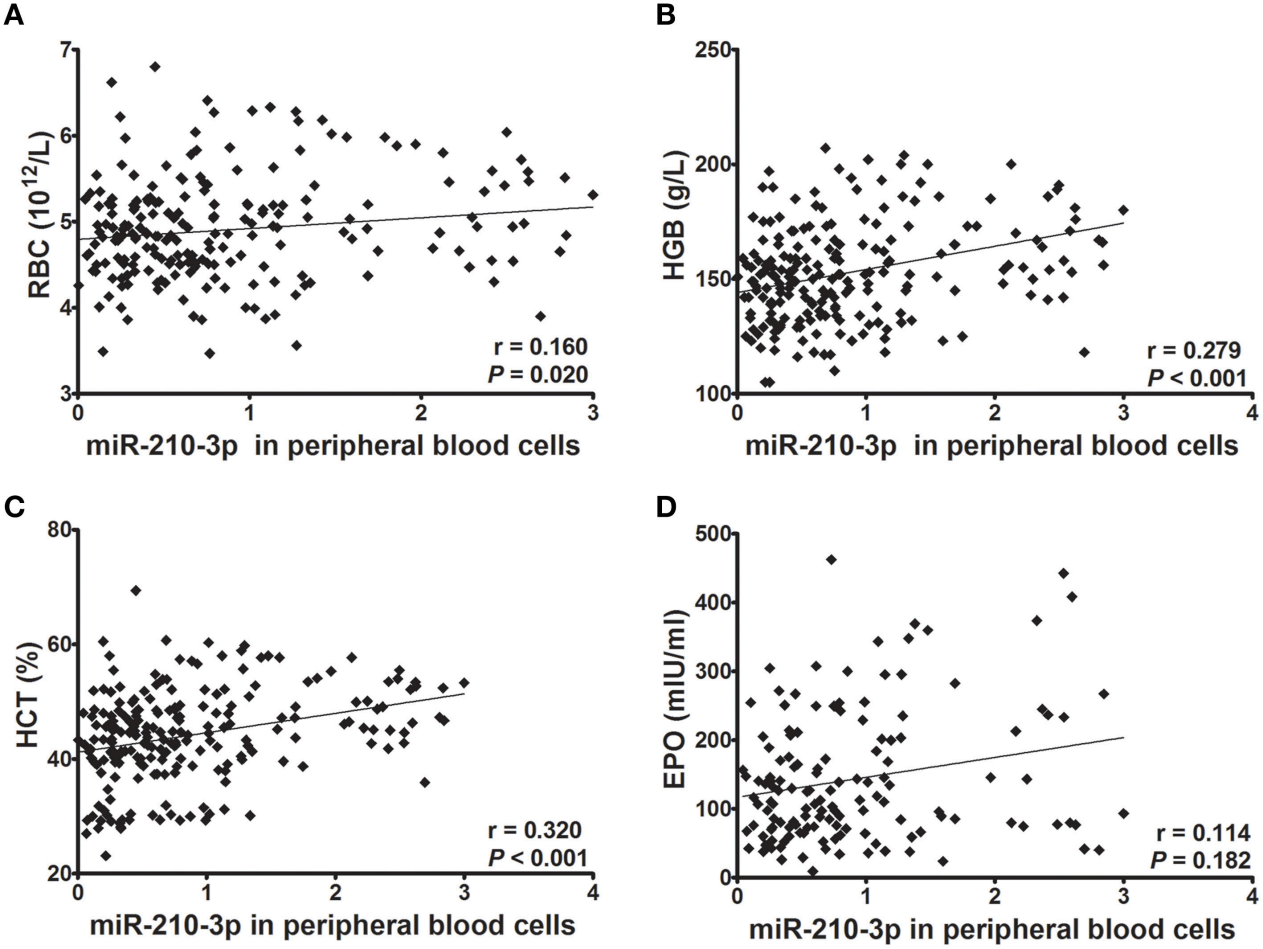
**The correlations of miR-210-3p expression levels in peripheral blood cells with hematological indices and plasma EPO concentrations (***n*** = 212). (A–D)** Correlations of miR-210-3p expression levels in blood cells with red blood cell count (RBC) and with hemoglobin (HGB), hematocrit (HCT), and plasma erythropoietin (EPO) values, calculated by Spearman's rank correlation analysis.

Next, we evaluated whether levels of miR-210-3p in plasma or in peripheral blood cells were related to hematological values and plasma EPO concentrations. Consequently, the absolute concentrations of miR-210-3p in plasma and the relative concentrations of miR-210-3p to U6 in blood cells showed significantly positive correlations with RBC, HGB and HCT values (Figures 2A–C, 3A–C) in all of the studied subjects. The plasma level of miR-210-3p also exhibited a significantly positive correlation with plasma EPO concentration (Figure 2D); however, no significant correlation was found between miR-210-3p levels in blood cells and plasma EPO concentrations (Figure 3D).

## Discussion

Hypoxia occurs in many physio-pathological processes, such as rapid tissue growth and acute and chronic ischemia, as well as at high altitudes. Previous data have indicated that hypoxia leaves a specific mark on miRNA profiles in a variety of cell types, with a critical contribution of HIF (Kulshreshtha et al., 2007). miR-210 is a well-known miRNA induced under hypoxic condition in several types of tissues and cells, and it contributes to cellular adaptation to hypoxic environments (Huang et al., 2010). Various of miR-210 targets have been identified, pointing to their roles in mitochondrial metabolism, angiogenesis, DNA damage response, apoptosis, and cell survival (Huang and Zuo, 2014). For example, the receptor tyrosine kinase ligand Ephrin-A3 (EFNA3) was identified as a miR-210 target for promoting the angiogenic properties of vascular endothelial growth factor (VEGF) (Fasanaro et al., 2008). E2F transcription factor 3 (E2F3) and fibroblast growth factor receptor-like1 (FGFRL1) were identified as miR-210 targets involved in cell cycle control (Nakada et al., 2011; Tsuchiya et al., 2011). In this study, we investigated the influence of high altitude hypoxic environments on miR-210-3p in human plasma and peripheral blood cells. We found that miR-210-3p in both plasma and blood cells was significantly higher in samples from either Tibet Han subjects or from Tibetan subjects than in samples from Nanjing Han subjects. To the best of our knowledge, this report is the first demonstrating that a high-altitude hypoxic environment has remarkable influences on human circulating miR-210-3p.

Since the discovery of circulating miRNA, their potential as molecular biomarkers for the diagnosis of various pathologic conditions has been explored. However, little is known about the origin of the extracellular miRNAs and what factors influence the levels of circulating miRNAs (Machado et al., 2015). Some studies have demonstrated that circulating miRNAs derive from a variety of sources. They derive not only from circulating blood cells but also from other injured cells affected by diseases (Chen et al., 2008). In this study, we observed a correlation in expression of miR-210-3p between the plasma and peripheral blood cells. But the origin of plasma miR-210-3p is not clear. Considering that hypoxia can affect the expression of miR-210-3p in a variety of cells, such as peripheral blood cells as observed in this study and endothelial cells (Fasanaro et al., 2008), we speculate that the increased plasma miR-210-3p might derive from these cells and other tissue cells in chronic hypoxic environments. The mechanism of miRNA secreted from cells to the blood circulation is not yet entirely clear. Active secretion by cells is a major source of circulating miRNAs; some miRNAs might also leak out of injured cells into the circulating blood (Ji et al., 2009; Iguchi et al., 2010; Ogawa et al., 2010; Wang et al., 2010). We speculate that miR-210-3p might mainly be secreted by the above-mentioned cells via cell-derived microvesicles or exosomes, or as microvesicles-free miRNAs into the circulation (Zen and Zhang, 2012) in response to hypoxic conditions, resulting in the increase of plasma miR-210-3p level. However, future studies are needed to clarify this issue.

Erythropoiesis, which is the process of erythroid production, is controlled by several factors, including oxygen levels. In the present study, we found that RBC, HGB, HCT values and plasma EPO concentrations of both men and women were significantly increased in the Tibet Han group compared with the Nanjing Han group. The HGB, HCT, and EPO values of both men and women, along with the RBC values in women, were also significantly higher in the Tibetan group than in the Nanjing Han group. The significance of elevated hematological indices in high altitude adaptation remains controversial. Some investigators believe that erythremia is a compensatory mechanism for hypoxemia at moderate altitudes and therefore that high levels of HGB represent a physiologic response and might be beneficial, but with increasing altitude, HGB and HCT can become excessive, losing their efficiency to serve the purpose of protecting the venous PO_2_ (León-Velarde et al., 2000). However, others believe that the polycythemia is pathological rather than indicative of adaptability (Garruto and Dutt, 1983), and even a slight increase in erythrocytosis will not have any physiological effect (Winslow et al., 1985). Previous studies have observed that the physiological response to low oxygen differs between Tibetans and individuals of low-altitude origin. For most individuals, acclimatization to low oxygen involves an increase in blood HGB levels. However, in Tibetans, the increase in HGB levels was limited. Further, the ability of Tibetans to adapt well to high altitude hypoxic environments might be a result of natural selection (Yi et al., 2010; Huerta-Sánchez et al., 2014). The same phenomenon was observed in our study, in which we compared these hematological parameters between the Tibet Han group and the Tibetan group and observed that those parameters were significantly increased in the Tibet Han men compared to the Tibetan men. Similarly, the levels of RBC, HGB, and HCT were also higher in Tibet Han women than in Tibetan women; however, the differences did not achieve statistical significance. Our results were agreement with previous studies showing that the HGB, HCT, and RBC were higher in men than in women for both Tibetan and Han subjects. However, the difference between men and women was less for Tibetans than for Han Chinese. Additionally, the difference was particularly striking among the Han men, who appeared to have much higher HGB values than the Tibetan men (Wu et al., 2014). It has been reported that post-menopausal Han women have higher HGB and HCT concentrations than pre-menopausal women (León-Velarde et al., 1997; Wu et al., 2014). Therefore, we speculated that ovarian hormones could have certain effects on the limit of the erythropoietic response to altitude and render Han women able to adapt to high altitude hypoxic environments better than Han men.

Recent studies are evidenced that the miRNAs are key regulators of all stages of hematopoiesis and hematopoietic disorders (Undi et al., 2013; Raghuwanshi et al., 2015). Several studies have shown significant role of miRNA in hematopoietic system markedly increased to more than 500 as listed in miRBase (www.mirbase.org) (Chen et al., 2004). Some miRNAs were reported to prevent the differentiation of early stage progenitor cells, or to regulate the terminal stages of hematopoietic development (Georgantas et al., 2007). miR-210-3p has also been reported to regulate the maturation and proliferation of early erythroid cells (Kosaka et al., 2008), and the expression of fetal g-globin genes and enucleation (Bianchi et al., 2009). A recent study demonstrated that hypoxia induced differentiation of both K562 and β-thalassemic erythroid progenitor cells, and this induction was at least in part mediated by miR-210 (Sarakul et al., 2013). In this study, we evaluated the correlations between miR-210-3p and hematological parameters, and we found that miR-210-3p in both plasma and blood cells showed strong and significant positive correlations with RBC, HGB, and HCT. In addition, plasma miR-210-3p concentrations were also found to be related to plasma EPO concentrations. Our study further demonstrated a relationship between erythropoiesis and miR-210-3p in hypoxia environments.

## Conclusions

We defined that the levels of miR-210-3p in plasma and blood cells were affected by high altitude hypoxic environments and that miR-210-3p might act as a circulating factor in response to hypoxic environments. Furthermore, we demonstrated strong positive relationships of circulating miR-210-3p with hematological parameters. Our results provided additional information regarding the molecular mechanisms of human adaptation to life at high altitude hypoxia, and miR-210-3p might contribute to this process.

## Author contributions

Conceived and designed the experiments: CZ, CYZ. Performed the experiments: YY, CW, WZ. Analyzed the data: YY. Contributed reagents/materials/analysis tools: PG, YS, JW. Wrote the paper: CZ, YY.

### Conflict of interest statement

The authors declare that the research was conducted in the absence of any commercial or financial relationships that could be construed as a potential conflict of interest.

## Supplementary material

Figure S1 **The Cq value of MIR2911 in plasma samples from the Nanjing Han, Tibet Han, and Tibetan groups**.

Figure S2 **Standard curve of miR-210-3p using synthetic miRNA**.

Figure S3 **The Cq values of U6 in peripheral blood cells samples from the Nanjing Han, Tibet Han, and Tibetan groups**.

Figure S4 **The relative concentrations of miR-210-3p in plasma samples from Nanjing Han, Tibet Han, and Tibetan groups**.
