# Supplementary figures and images for: Elevation of Circulating miR-210-3p in High-Altitude Hypoxic Environment
Source: Front Physiol. 2016 Mar 8;7:84. doi: 10.3389/fphys.2016.00084 (PMC4781857; doi:10.3389/fphys.2016.00084)

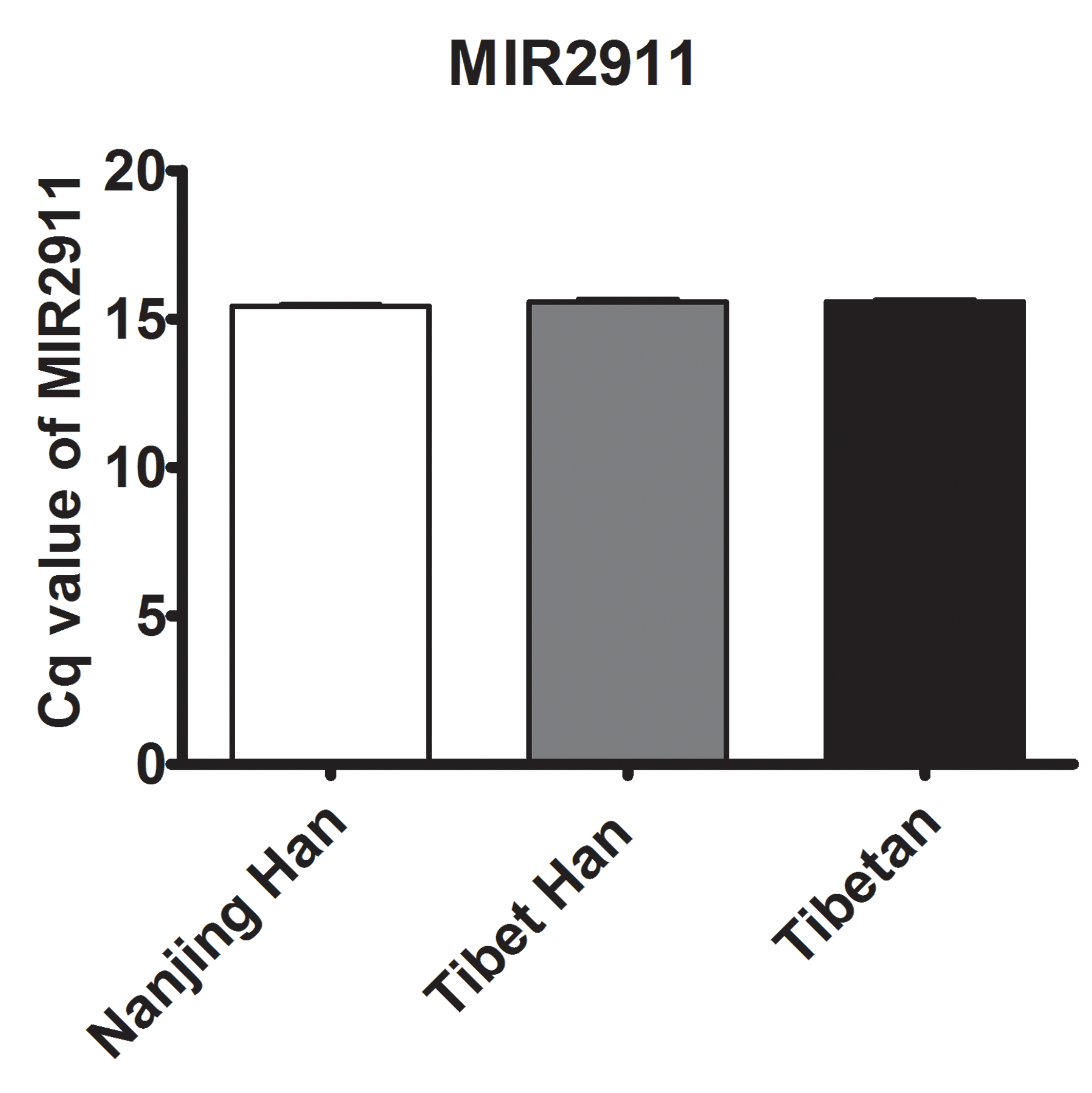

Supplement: Figure S1 — The Cq value of MIR2911 in plasma samples from the Nanjing Han, Tibet Han, and Tibetan groups. [file Image1.TIF]

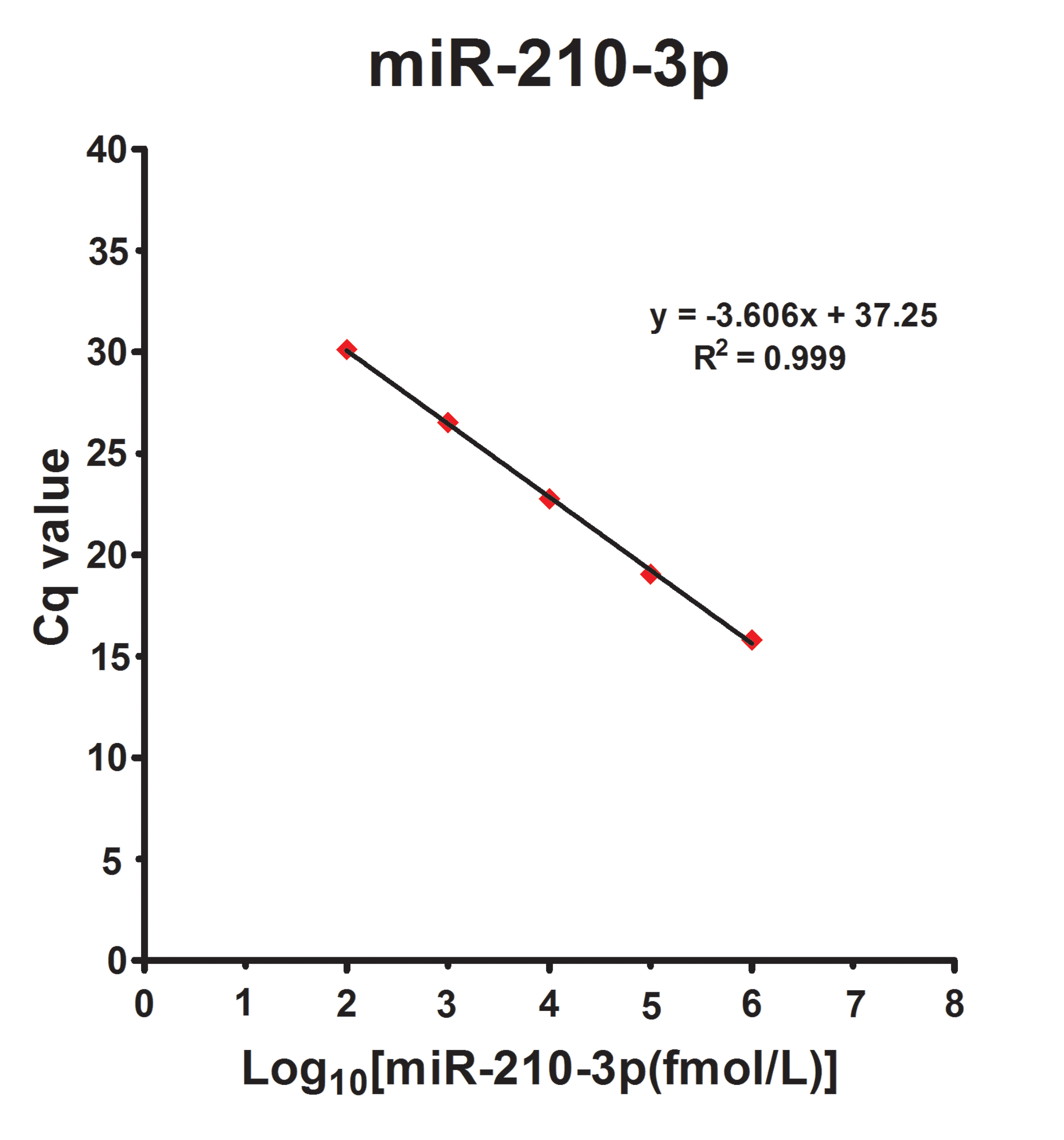

Supplement: Figure S2 — Standard curve of miR-210-3p using synthetic miRNA. [file Image2.TIF]

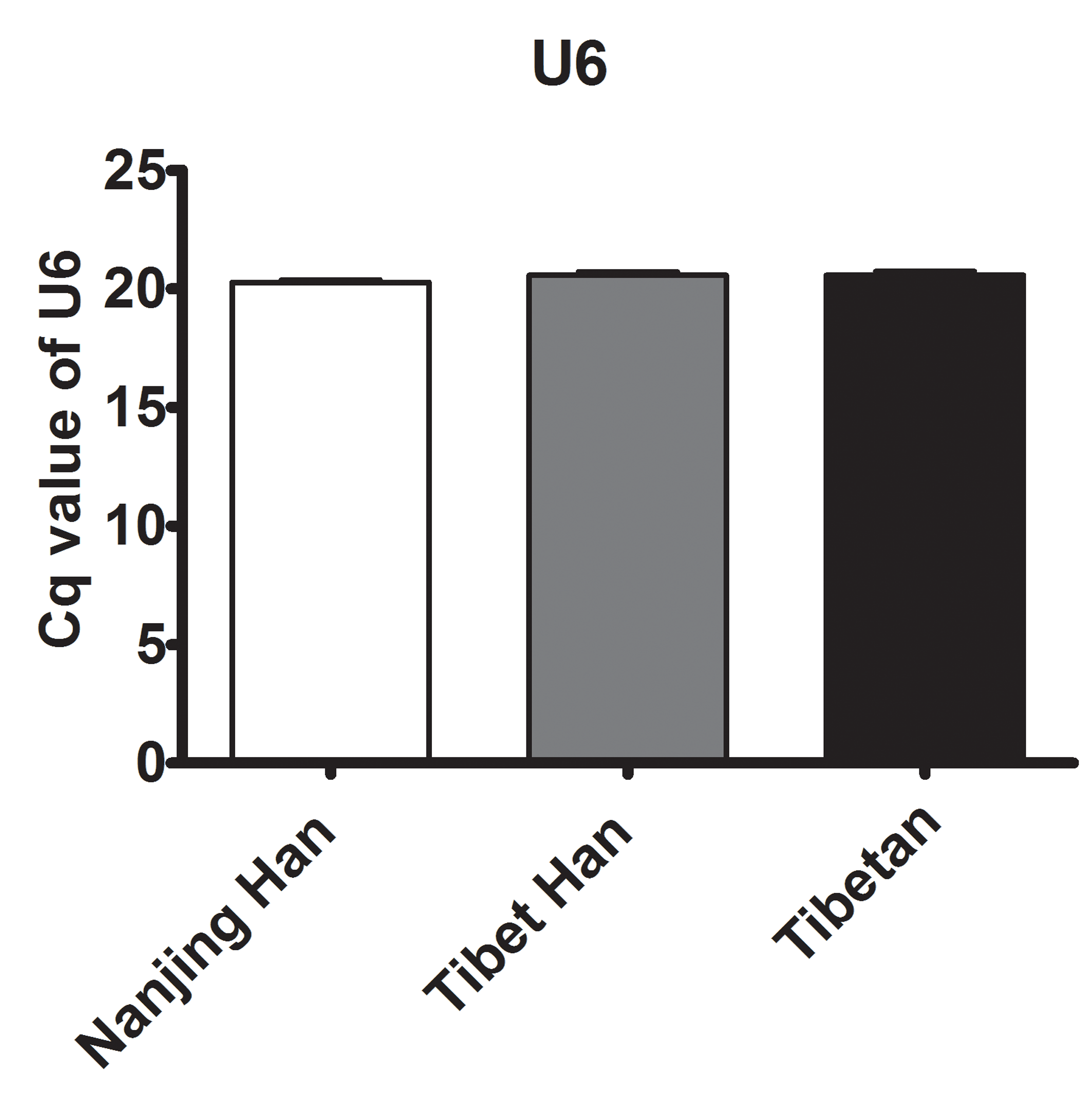

Supplement: Figure S3 — The Cq values of U6 in peripheral blood cells samples from the Nanjing Han, Tibet Han, and Tibetan groups. [file Image3.TIF]

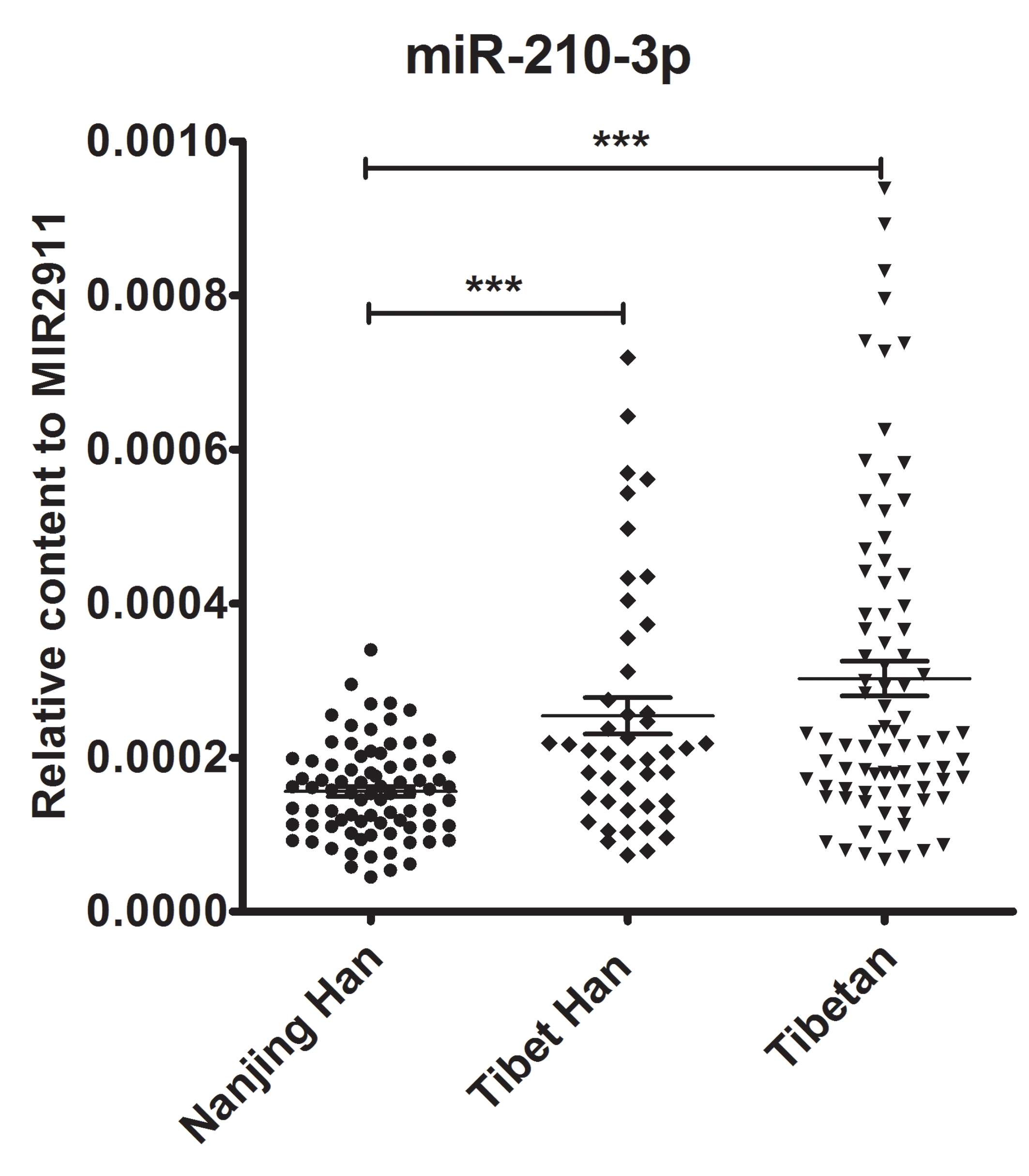

Supplement: Figure S4 — The relative concentrations of miR-210-3p in plasma samples from Nanjing Han, Tibet Han, and Tibetan groups. [file Image4.TIF]
